# Perirenal Hematoma and Graft Malposition Requiring Surgical Repositioning After Transplant Tourism: A Case Report

**DOI:** 10.1002/iju5.70254

**Published:** 2026-08-25

**Authors:** Kazuki Miyazaki, Shunta Hori, Mitsuru Tomizawa, Kuniaki Inoue, Tomoharu Iwao, Yasushi Nakai, Makito Miyake, Tatsuo Yoneda

**Affiliations:** ^1^ Department of Urology Nara Medical University Kashihara, Nara Japan

**Keywords:** exploratory laparotomy, graft malposition, kidney transplantation, perirenal hematoma, transplant tourism

## Abstract

**Introduction:**

Patients returning after transplant tourism often have incomplete perioperative information, complicating postoperative management and clinical decision‐making.

**Case Presentation:**

A 72‐year‐old Japanese man with end‐stage kidney disease secondary to diabetic nephropathy underwent living‐donor kidney transplantation abroad. After returning to Japan, he developed decreased urine output and lower back pain. Contrast‐enhanced computed tomography revealed the transplanted kidney in the subcutaneous tissue of the right lower abdomen, with perirenal gas/fluid collection and a partially non‐enhancing lesion in the renal parenchyma. Because bowel injury could not be excluded, exploratory laparotomy was performed. Intraoperatively, the graft was identified in the subcutaneous layer with surrounding hematoma and was repositioned into the subfascial iliac fossa. Postoperatively, graft function remained stable without pathological abnormalities.

**Conclusion:**

Transplant tourism may involve substantial deviation from standard surgical practice and poses significant clinical and ethical challenges for physicians.

## Keynote Message

Patients returning after kidney transplantation abroad often present with incomplete perioperative information, making postoperative management challenging. Careful assessment of graft anatomy together with all available operative and donor‐recipient information is essential for clinical decision‐making. When nonstandard graft anatomy or visceral injury cannot be excluded, prompt imaging assessment and timely surgical exploration should be considered. This case highlights the importance of a systematic approach to the evaluation and management of patients returning after transplant tourism.

## 1 Introduction

In Japan, the shortage of deceased‐donor kidney transplantation has prolonged waiting times, leading some patients to seek transplantation abroad, known as transplant tourism. According to the Declaration of Istanbul, transplant tourism and organ commercialism are unethical practices [1, 2]. Despite these international efforts, transplant tourism continues, particularly in regions with severe organ shortages.

The problems associated with transplant tourism extend beyond ethical concerns. Patients who undergo transplantation abroad frequently return with incomplete medical records, making it difficult to assess donor characteristics, perioperative management, and immunosuppressive protocols [3, 4]. This lack of information can delay the diagnosis of complications and complicate postoperative management. Outcomes after transplant tourism have been reported to be inferior to those after standard transplantation, with higher rates of rejection, infection, and graft loss [5, 6].

In addition to these challenges, physicians can encounter unexpected surgical findings that deviate from standard transplantation practices. In such cases, clinical decision‐making becomes particularly difficult because management must be determined under conditions of substantial uncertainty and limited information. However, detailed reports that focus on nonstandard graft positioning and the associated decision‐making processes remain scarce.

Herein, we report a patient who underwent living‐donor kidney transplantation abroad and subsequently developed a perirenal hematoma with an unusually positioned graft that required surgical repositioning. This case highlights not only the medical risks associated with transplant tourism, but also the clinical and ethical challenges encountered by physicians who provide postoperative care under such conditions.

## 2 Case Presentation

A 72‐year‐old Japanese man with end‐stage kidney disease secondary to diabetic nephropathy underwent living‐donor kidney transplantation in Pakistan. No operative note, referral letter, or perioperative summary was available. The donor‐recipient relationship, compatibility, ABO/HLA matching, crossmatch results, ischemia times, reconstruction details, perioperative rejection episodes, and early renal function trends were unknown. The documented immunosuppressive regimen consisted of cyclosporine, mycophenolate mofetil, and prednisolone. He returned to Japan on postoperative Day 7 with a pelvic drain in place and subsequently developed decreased urine output and lower back pain. Computed tomography revealed the graft in the subcutaneous tissue of the right lower abdomen, and he was referred to our institution on postoperative Day 12.

**FIGURE 1:**
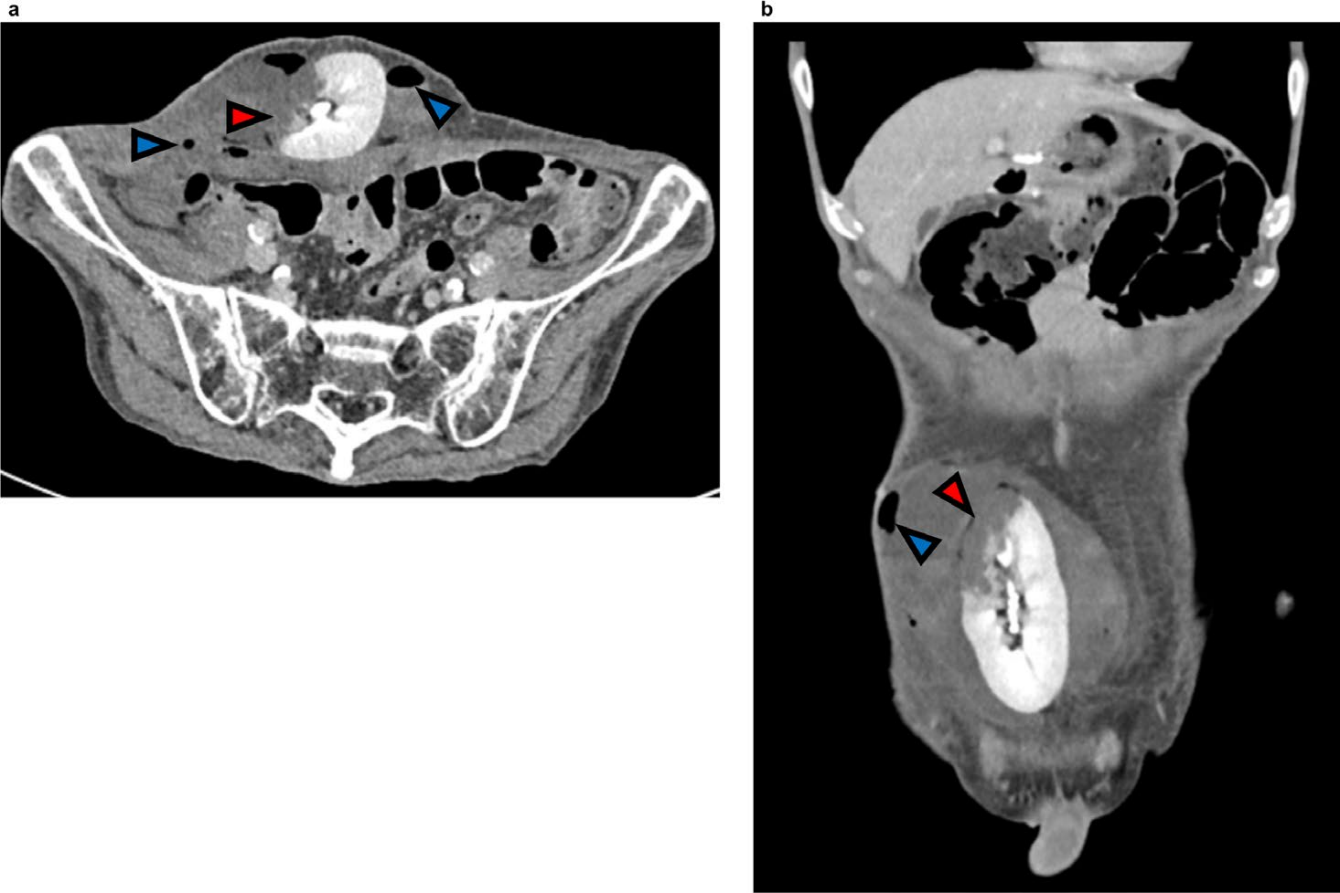
Contrast‐enhanced computed tomography at presentation. (a) Axial image showing the transplanted kidney located in the subcutaneous tissue of the right lower abdomen. Blue arrowheads indicate perirenal gas, and the red arrowhead indicates a partially non‐enhancing ischemic area within the renal parenchyma. (b) Coronal image demonstrating subcutaneous graft malposition with surrounding gas and fluid collection; blue arrowhead indicates gas, and the red arrowhead indicates a focal ischemic area.

Physical examination showed right lower abdominal bulging and tenderness. Laboratory findings demonstrated hyperbilirubinemia and hepatobiliary enzyme elevation, while renal function was preserved (serum creatinine, 0.78 mg/dL). The cyclosporine trough level was markedly elevated at 844.4 ng/mL. Contrast‐enhanced computed tomography showed the graft in the subcutaneous tissue of the right lower abdomen with surrounding gas/fluid collection and a partially non‐enhancing lesion (Figure 1a,b). Although bowel evisceration was not identified, bowel injury or herniation could not be excluded; therefore, exploratory laparotomy was performed.

**FIGURE 2:**
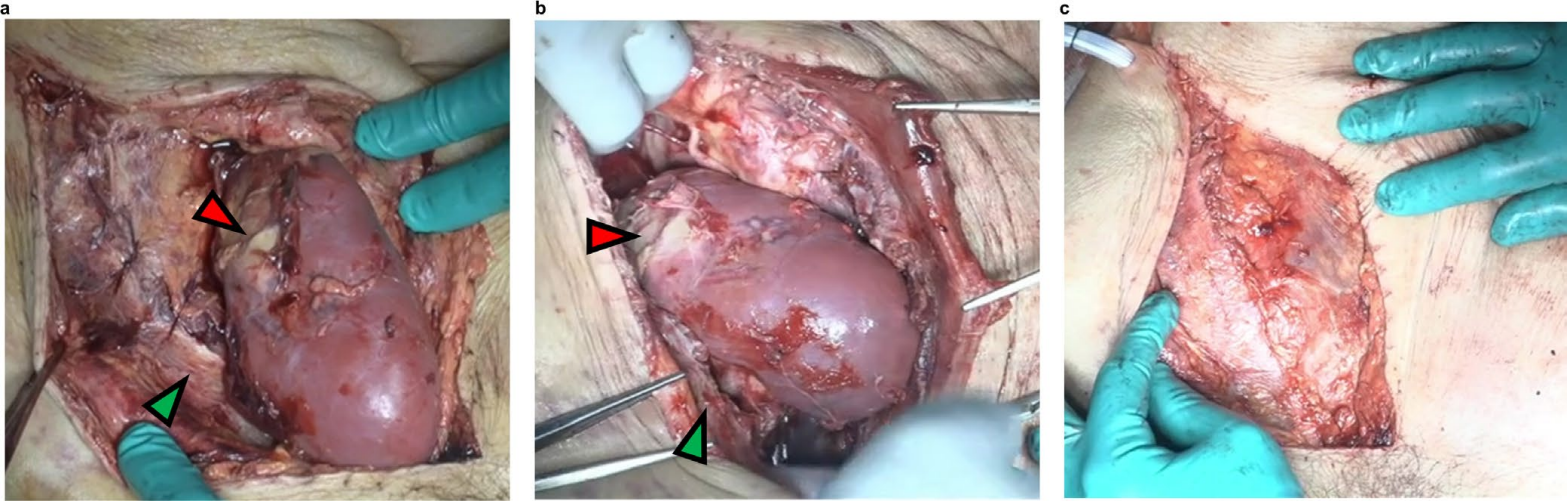
Intraoperative findings. (a, b) Transplanted kidney was identified in the subcutaneous space; green arrowheads indicate the fascial layer, suggesting that the graft was positioned in the subcutaneous space above a closed fascial layer, and red arrowheads indicate the focal ischemic area on the graft surface. (c) After repositioning of the graft into the iliac fossa, the wound was closed.

Intraoperatively, the graft was identified in the subcutaneous layer rather than the usual iliac fossa (Figure 2a). No bowel injury was detected. Perirenal hematoma and fluid collection were observed around the graft.

**FIGURE 3:**
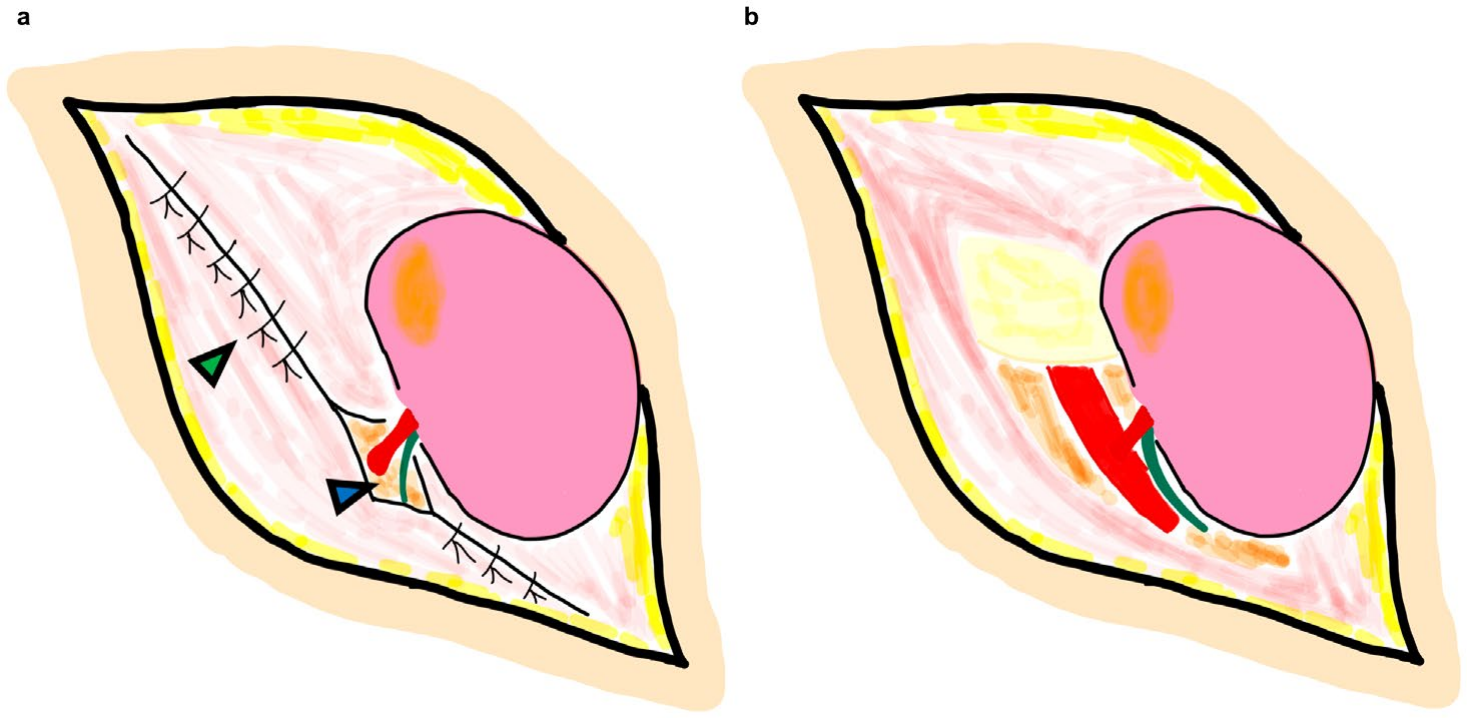
Schematic illustration of the intraoperative findings and surgical revision. (a) The renal allograft was located superficial to the abdominal fascia and fixed to the fascia with interrupted sutures, except at its caudal aspect. The graft hilar structures and ureter passed through this unfixed caudal opening. A hematoma was present around the graft, and the upper pole appeared yellowish, corresponding to the ischemic area observed on contrast‐enhanced computed tomography. The transplant renal vein could not be identified intraoperatively. The green arrow indicates the fascia, and the blue arrow indicates the retroperitoneal space through which the transplanted ureter and blood vessels entered. (b) After removal of the fascial fixation sutures, the transplant renal artery, external iliac artery, and ureter were identified. The fascia was further dissected to create sufficient space, and the renal allograft was repositioned beneath the fascia without tension or compression. Intraoperative ultrasonography confirmed adequate graft perfusion after fascial closure.

The graft was fixed to the fascia with interrupted sutures except at its caudal aspect, where the hilar structures and ureter passed. After removal of the fixation sutures, the transplant renal artery, external iliac artery, and ureter were identified; however, the transplant renal vein could not be identified. To avoid vascular injury, circumferential dissection of the pedicle was not performed. Following additional fascial and peritoneal dissection, the graft was repositioned beneath the fascia without torsion or compression (Figure 2b,c). The intraoperative anatomy and surgical revision are illustrated in Figure 3a,b.

**FIGURE 4:**
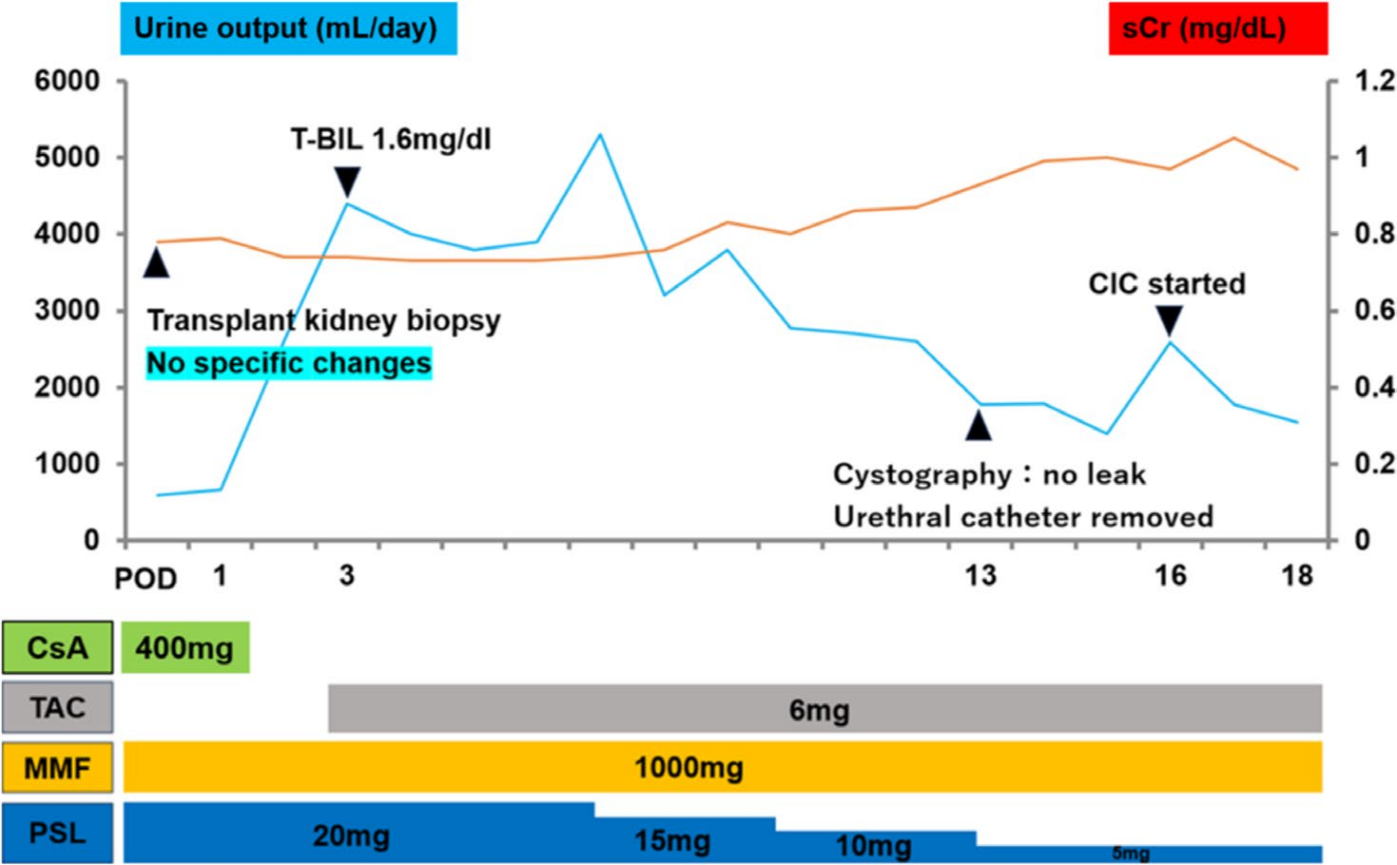
Postoperative course. Graft biopsy showed no specific pathological abnormalities, and urine output and serum creatinine remained stable during follow‐up. CIC, clean intermittent catheterization; CsA, cyclosporine A; MMF, mycophenolate mofetil; POD, postoperative day; PSL, prednisolone; TAC, tacrolimus; T‐BIL, total bilirubin.

The postoperative course was uneventful. A graft biopsy showed no acute rejection or other pathological abnormalities. Serum creatinine remained stable at approximately 0.7–1.0 mg/dL. Hepatobiliary enzyme abnormalities improved after conversion from cyclosporine to tacrolimus. Cystography showed no urinary leakage. Clean intermittent catheterization was initiated for voiding dysfunction, and the patient was discharged on postoperative day 18 with preserved graft function (Figure 4).

## 3 Discussion

The exact circumstances leading to this graft positioning remain unclear because operative records were unavailable. However, the graft location superficial to the fascial layer strongly suggested deviation from accepted surgical practice. Because circumferential dissection of the vascular pedicle was intentionally avoided to prevent vascular injury during revision surgery, the course of the transplant renal vein could not be fully delineated intraoperatively. Graft malposition or torsion has mainly been reported in intraperitoneal grafts [7], whereas such complications are rare in retroperitoneal placement [8]. To our knowledge, subcutaneous graft positioning has not been previously reported. In addition, the coexistence of a perirenal hematoma and focal ischemic lesion further increased the risk of graft compromise. In the present case, imaging findings were insufficient to exclude bowel injury or herniation, and the lack of operative information further increased diagnostic uncertainty. Exploratory laparotomy was therefore necessary to establish a diagnosis and prevent potentially catastrophic complications. This case highlights the importance of timely surgical intervention based on limited information in patients returning after transplant tourism.

The absence of perioperative information represents a major barrier to postoperative management in transplant tourism. Previous studies have reported that patients undergoing transplantation abroad frequently return with incomplete medical records, making it difficult to assess donor characteristics, perioperative events, and immunosuppressive protocols [3, 4]. In the present case, the adequacy of pretransplant evaluation remained uncertain despite the patient's history of angina pectoris and gastric cancer. Current guidelines emphasize careful assessment of cardiovascular disease, malignancy, infection, and immunological compatibility before kidney transplantation [9, 10]. Outcomes after transplant tourism have been reported to be inferior to those after standard transplantation, with increased risks of rejection, infection, and graft loss [5, 6, 11].

Another notable finding was the markedly elevated blood cyclosporine concentration accompanied by hepatobiliary enzyme abnormalities. Possible explanations include inappropriate dosing, lack of therapeutic drug monitoring, and drug interactions. Improvement in liver function after conversion to tacrolimus supported the possibility of cyclosporine‐related toxicity, highlighting the risks associated with inadequate perioperative management in transplant tourism.

This case also raised important ethical concerns. Although transplant tourism is considered unethical according to the Declaration of Istanbul [1, 2], physicians are still required to provide appropriate postoperative care.

When imaging cannot exclude bowel injury, herniation, or graft compromise, prompt surgical exploration should be considered. This case also underscores the importance of assessing graft anatomy and reviewing all available perioperative information for the safe management of patients returning after transplant tourism. Although transplant tourism remains ethically unacceptable, physicians must provide safe and timely care under these challenging circumstances.

## Consent

Written informed consent for publication of this case report was obtained from the patient himself.

## Conflicts of Interest

The authors declare no conflicts of interest.

## Data Availability

The data that support the findings of this study are available on request from the corresponding author. The data are not publicly available due to privacy or ethical restrictions.

## References

[1] Declaration of Istanbul Custodian Group , “The Declaration of Istanbul on Organ Trafficking and Transplant Tourism (2018 Edition),” Transplantation 103, no. 2 (2019): 218–219. 30681644 10.1097/TP.0000000000002540

[2] D. A. Budiani‐Saberi and F. L. Delmonico , “Organ Trafficking and Transplant Tourism: A Commentary on the Global Realities,” American Journal of Transplantation 8 (2008): 925–929. 18416734 10.1111/j.1600-6143.2008.02200.x

[3] A. Altheaby , K. Owaidah , A. Alotaibi , et al., “Graft and Patient Outcomes of Kidney Transplant Tourism: A Single‐Center Experience,” Avicenna Journal of Medicine 12 (2022): 120–126. 36092382 10.1055/s-0042-1750715 PMC9458345

[4] M. Tawhari and M. Radwi , “A Three‐Year Experience With Overseas Kidney Transplantation in a Tertiary Transplant Center in Saudi Arabia,” Cureus 14 (2022): e23988. 35419250 10.7759/cureus.23988 PMC8994614

[5] M. M. Al Bugami , F. E. Al Otaibe , A. M. Alabadi , K. Hamawi , and K. Bel'eed‐Akkari , “Transplant Tourism Following the Declaration of Istanbul: Poor Outcomes and Nephrologist Dilemma,” Nephrology (Carlton) 23 (2018): 1139–1144. 29030994 10.1111/nep.13181

[6] J. Gill , B. R. Madhira , D. Gjertson , G. Lipshutz , J. M. Cecka , and P. T. Pham , “Transplant Tourism in the United States: A Single‐Center Experience,” Clinical Journal of the American Society of Nephrology 3 (2008): 1820–1828. 18922987 10.2215/CJN.02180508 PMC2572286

[7] A. Lucewicz , A. Isaacs , R. D. M. Allen , V. W. T. Lam , S. Angelides , and H. C. C. Pleass , “Torsion of Intraperitoneal Kidney Transplant,” ANZ Journal of Surgery 82 (2012): 299–302. 22507693 10.1111/j.1445-2197.2011.05792.x

[8] M. Sosin , W. Lumeh , and M. Cooper , “Torsion of the Retroperitoneal Kidney: Uncommon or Underreported?,” Case Reports in Transplantation 2014 (2014): 561506. 24551473 10.1155/2014/561506 PMC3914369

[9] S. J. Chadban , C. Ahn , D. A. Axelrod , et al., “KDIGO Clinical Practice Guideline on the Evaluation and Management of Candidates for Kidney Transplantation,” Transplantation 104, no. 4 Suppl 1 (2020): S11–S103. 32301874 10.1097/TP.0000000000003136

[10] Japan Organ Transplant Network , “Recipient Selection Criteria for Kidney Transplantation,” Revised March 2026. [in Japanese].

[11] N. Ivanovski , J. Masin , I. Rambabova‐Busljetic , et al., “The Outcome of Commercial Kidney Transplant Tourism in Pakistan,” Clinical Transplantation 25 (2011): 171–173. 20626425 10.1111/j.1399-0012.2010.01299.x

